# Educational Case: Acetaminophen hepatotoxicity: Pathophysiology and evaluation of acute liver failure

**DOI:** 10.1016/j.acpath.2024.100146

**Published:** 2024-09-09

**Authors:** Hunter L. Monroe, Tony El Jabbour

**Affiliations:** Department of Pathology, Anatomy, and Laboratory Medicine, West Virginia University, Morgantown, WV, USA

**Keywords:** Acetaminophen, Acute liver failure, Hepatobiliary, Liver toxins, Organ system pathology, Pathology competencies

The following fictional case is intended as a learning tool within the Pathology Competencies for Medical Education (PCME), a set of national standards for teaching pathology. These are divided into three basic competencies: Disease Mechanisms and Processes, Organ System Pathology, and Diagnostic Medicine and Therapeutic Pathology. For additional information, and a full list of learning objectives for all three competencies, see https://www.journals.elsevier.com/academic-pathology/news/pathology-competencies-for-medical-education-pcme.^1^

## Primary objective

Objective HB2.2: Acetaminophen Toxicity. Describe the clinicopathologic features of excessive acetaminophen ingestion focusing on biochemical pathways and short-and long-term complications.

Competency 2: Organ System Pathology; Topic HB: Hepatobiliary; Learning Goal 2: Liver Toxins.

## Patient presentation

A 35-year-old man is brought to the emergency room by his wife, who provided the medical history, for complaints of severe right upper quadrant (RUQ) pain, nausea, and fatigue that began the evening prior. At presentation, he is falling in and out of sleep and unable to answer questions in a coherent manner. When asked about the history surrounding his presentation, his wife states that he was resting comfortably in his recliner when he began to complain about sudden RUQ pain and does not recall any preceding events that may have incited these symptoms. Within several hours of the aforementioned initial symptoms, his wife stated that he additionally started “acting strange”. His wife further clarifies that he has mentioned having some suicidal ideation in the past but has never made any attempts to her knowledge. Social history is pertinent for drinking 3 glasses of whiskey after coming home from his desk job on most days. Additionally, he has never traveled out of the country before. The patient's family history includes only type 2 diabetes mellitus for his mother and hypertension and myocardial infarction regarding his father.

## Diagnostic findings, Part 1

Physical exam reveals a fatigued and stuporous male patient. Vital signs include a temperature of 97 °F, heart rate of 102 bpm, blood pressure of 80/55 mmHg, and respiratory rate of 17 breaths per minute. Findings of the abdominal exam include hepatomegaly upon palpation and RUQ tenderness. A yellow tint is noted on his skin and sclera upon inspection. Cardiovascular exam reveals regular heart rate and rhythm, no additional sounds, and no peripheral edema. Lungs are clear to auscultation bilaterally with no increased work of breathing. The patient's head appears normocephalic and atraumatic. Neurologic examination reveals that the man is disoriented to person, place, time, and situation but mildly responsive to verbal stimuli and a symmetric flapping tremor of his hands is conspicuous upon extension of his wrists. Bruises, which his wife does not recall seeing until this examination, are present on his forearm and back. The patient's mental status declines and he becomes comatose shortly after the examination is completed.

Routine laboratory assays are ordered for this patient (see Table 1).

**Table 1:** Laboratory assays.

Test	Value	Flag	Reference range
**Basic metabolic panel with blood ammonia**			
Sodium (mEq/L)	136		136–145
Potassium (mEq/L)	4.0		3.5–5.0
Chloride (mEq/L)	98		95–105
Bicarbonate (mEq/L)	15	Low	22–26
Blood Urea Nitrogen (mg/dL)	24	Elevated	6–20
Serum Creatinine (mg/dL)	3.7	Elevated	0.6–1.3
Serum Glucose (mg/dL)	59	Low	70–100
Aspartate Aminotransferase (U/L)	10,043	Elevated	8–33
Alanine Aminotransferase (U/L)	10,051	Elevated	7–55
Blood Ammonia (μg/dL)	104	Elevated	15–45
**Platelets and coagulation panel**			
Platelets (×10^3^/mm^3^)	70	Low	150–400
Prothrombin Time (s)	104	Elevated	11–13.5
Activated Partial Thromboplastin Time (s)	89	Elevated	30–40
International Normalized Ratio (INR)	6.7	Elevated	<1.1
**Arterial blood gases with lactic acid**			
pH	7.23	Low	7.35–7.45
PaCO_2_ (mmHg)	28	Low	35–45
PaO_2_ (mmHg)	98		75–100
Lactic Acid (mg/dL)	28.7	Elevated	4.5–19.8

## Questions/discussion points, Part 1

### What is the most likely diagnosis based on the patient's clinical and laboratory findings?

The patient's RUQ pain and jaundice are suggestive of acute liver injury. However, the development of hepatomegaly and multisystemic symptoms implies that more severe injury has occurred. Elevated blood ammonia in combination with stupor, gross disorientation, and flapping tremor (asterixis) are consistent with hepatic encephalopathy. Hepatic encephalopathy is often graded according to the West Haven Criteria, which specifically address the cognitive symptoms of the disease and consist of grade I (inattention with subtle personality changes), II (a triad of disorientation to time, inappropriate behavior, and lethargy), III (stupor with responsiveness to stimuli and disorientation to place and situation), and IV (coma).^2^

Based on this criteria, the patient's hepatic encephalopathy is initially grade III before progressing to grade IV after examination. The elevated coagulation assays as well as new-onset bruising on the extremities are emblematic of a coagulopathy, a sequela of failing liver synthetic activity. Elevated serum creatinine in the absence of chronic kidney disease indicates that acute renal injury may have taken place. Finally, the patient's arterial blood gases with elevated lactic acid confirm that metabolic acidosis has occurred.

Thus, the patient's findings are consistent with acute liver failure (ALF), which is defined as acute liver injury with symptoms indicative of severe damage to hepatocytes including INR ≥1.5, encephalopathy, and liver transaminases >3× upper limit of normal without preexisting liver disease.^3^ Though there is no official temporal cutoff for the diagnosis of ALF, many clinicians quantify ALF as the development of severe liver injury symptoms less than 26 weeks after the onset of acute liver injury.^3^, ^4^, ^5^

### What are the major etiologies of acute liver failure?

Drug-induced liver injury (DILI) accounts for the majority of ALF cases in developed nations.^5^^,^^6^ DILI-associated ALF may be subdivided into dose-dependent DILI (dDILI) or idiosyncratic DILI (iDILI) patterns. dDILI-associated ALF is predictable and easily reproducible. The most common cause of dDILI is acetaminophen overdose, which most often arises 8–12 h after ingestion.^4^, ^5^, ^6^ Ingestion of toxins including alpha-amanitin and phalloidin produced by the death cap mushroom (*Amanita phalloides*) and carbon tetrachloride, an industrial agent used as a refrigerant and solvent for waxes and varnishes, may serve as further etiologies of dDILI-associated ALF.^6^

Conversely, iDILI-associated ALF behaves in a less predictable and non-dose-responsive manner.^7^ The most commonly reported etiologies of iDILI-associated ALF include antituberculous medications such as isoniazid, anesthetics including halothane and enflurane, statins, and anti-epileptics such as phenytoin and valproate. Herbal and drug supplements such as green teas, black cohosh, and various weight loss products deserve special mention as they now account for up to 20% of iDILI-associated ALF in the US, are unregulated by the U.S. Food and Drug Administration (USFDA), and are associated with higher mortality rates than other etiologies (34% vs 17% for prescription medications).^7^

Infection by hepatotrophic viruses remains the most common cause of ALF in developing nations. A rapid presentation (less than 1–4 weeks) is most frequently associated with hepatitis A and E. Hepatitis A is most commonly contracted through poor sanitary practices or fecal-oral transmission and has a worldwide distribution but is most common in developing nations. Hepatitis E is endemic to North Africa and Southern Asia and associated with contaminated water and food. ALF due to hepatitis E is rare and occurs predominantly in females in the third trimester of pregnancy.^6^ Hepatitis B accounts for 8% of ALF cases in developed countries, of which up to 2/3 are due to decompensation of a chronic infection, with transmission via sexual contact or exposure to infected blood from needles or razors.^5^^,^^6^ Unlike ALF associated with other hepatotropic viruses, hepatitis B often has a more delayed onset (1–4 weeks).^6^ Notably, the risk of ALF with hepatitis B infection increases 2–5-fold if hepatitis D coinfection has occurred.^6^ Other viruses less commonly associated with ALF include herpes simplex virus, cytomegalovirus, Epstein-Barr virus, varicella zoster, and adenovirus.^6^

Additional etiologies of ALF include ischemic hepatitis, hepatic venous outflow obstruction (Budd-Chiari Syndrome), autoimmune hepatitis, and Wilson disease.^3^^,^^6^

### What additional historical details may be important to elucidate from the patient's wife to narrow the differential diagnosis?

Additional history asked of the patient's wife should include the current or past use of medications, especially acetaminophen, anti-epileptics, and any drugs used for the treatment of tuberculosis. Other historical details to note include recent surgeries where a hepatotoxic-inhaled anesthetic such as halothane was administered, use of herbal products and supplements, consumption of wild mushrooms, a known history of viral hepatitis, the presence of congestive heart failure or other conditions predisposing to ischemia, and a family history of thrombotic disorders and Wilson disease.

## Diagnostic findings, Part 2

The patient's wife clarifies that her husband uses acetaminophen and loratadine regularly for headaches and seasonal allergies, respectively, and keeps them in a medicine cabinet within their bathroom. He does not have a history of epilepsy and has never contracted tuberculosis. Furthermore, he does not advocate the use of herbal products and doesn't take any dietary supplements. The patient's wife explains that he does not partake in outdoor recreational activities and doesn't eat mushrooms to her knowledge. He has never been diagnosed with viral hepatitis as far as she is aware. He has no history of congestive heart failure. She is not aware of a history of Wilson disease or thrombotic disorders in her husband's family.

Upon further questioning regarding her husband's acetaminophen use, the wife states he was in the bathroom longer than usual and his symptoms started shortly after this time.

Subsequently, a laboratory panel to elucidate the likely cause of ALF in this patient is ordered (see Table 2).

**Table 2:** Acute liver failure panel.

Test	Value/Result	Flag	Reference range
Anti-HAV IgM	Negative		N/A
HBsAg	Negative		N/A
Anti-HBc IgG	Negative		N/A
Anti-HBs IgG	Positive	Evaluate in context of other serologies and clinical characteristics	N/A
Anti-Smooth Muscle Antibody (ASMA)	Negative		<1:100
Anti-Liver Kidney Microsome 1 (ALKM1)	Negative		<20U
Anti-Nuclear Antibody (ANA)	Negative		<1:40
Ceruloplasmin (mg/dL)	24		14–40
Serum Copper (μg/dL)	95		70–140
Serum Acetaminophen (μg/dL)	41.2	Elevated	<6.63

## Questions/discussion points, Part 2

### Explain the significance of the patient's etiology-specific history and labs

The patient's lack of travel to countries with endemic hepatitis A and E renders viral etiologies less likely. The patient's negative anti-HBc and positive anti-HBs confirm that the patient has received vaccination for hepatitis B. Subacute DILI due to herbal products or dietary supplements is unlikely to be the cause of ALF in this patient. No history of heritable liver diseases such as Wilson disease is known and this is reflected in the patient's normal ceruloplasmin and serum copper values. All autoimmune hepatitis-related antibody assays are negative, effectively ruling out autoimmune hepatitis. Ultimately, the patient's elevated serum acetaminophen at approximately 22 h and history of depression confirm acetaminophen toxicity has taken place.

### Describe the mechanism of action of acetaminophen and the epidemiology of acetaminophen overdoses

Acetaminophen, also called paracetamol, is an analgesic and antipyretic that is believed to exert its effects through the selective inhibition of brain cyclooxygenase-2 (COX-2) and modulation of the hypothalamus.^8^ Acetaminophen is the most widely used over-the-counter (OTC) product in the world.^9^ Though safe at therapeutic doses of <4 g/day in adults and <50–75 mg/kg/day in children,^10^ overdoses, especially those exceeding 10 g/day, may cause liver injury and are attributed to 42–46% of all cases of ALF, thus making it the most common cause of ALF in the West.^10^^,^^11^ Unintentional overdoses, which account for >50% of cases of acetaminophen-related ALF, are often due to the use of acetaminophen-containing combination products of which it has been reported that up to 56.7% and 13.6% of overdoses are due to products containing opioids and diphenhydramine, respectively.^9^

### What is the pathophysiology of acetaminophen hepatotoxicity?

At therapeutic doses, approximately 90% of acetaminophen is metabolized by phase II conjugation enzymes, UDP-glucuronosyltransferase (UGT) and sulfotransferase (SULT), to nontoxic metabolites that are excreted in urine while the remaining 10% is metabolized by CYP2E1 to N-acetyl-p-benzoquinone imine (NAPQI), which is detoxified by the antioxidant glutathione (GSH) to nontoxic mercaptate and excreted in urine. Following supratherapeutic doses, phase II enzymes are saturated and more NAPQI is synthesized by CYP2E1 than can be reduced by GSH. Excess NAPQI then covalently binds the sulfhydryl (–SH) groups of mitochondrial proteins to form NAPQI-protein adducts that interfere with the activity of complexes I and II of the electron transport chain (ETC).^12^ Disruption of the ETC results in the massive leakage of electrons, which bind with oxygen to form the reactive oxygen species (ROS) superoxide radicals, which may be metabolized by manganese superoxide dismutase (SOD) to hydrogen peroxide (H_2_O_2_) and finally to the highly reactive hydroxide (OH•) radical, or react with endogenous nitric oxide to form peroxynitrite, a reactive nitrogen species (RNS). Interactions of these ROS and RNS with cellular biomolecules result in oxidation or nitrosylation of membrane lipids, mitochondrial proteins, and DNA, further leading to cell death by necrosis.^11^^,^^12^ Because CYP450 levels are at their highest in zone 3 (perivenular) hepatocytes, more NAPQI is synthesized at this site, elucidating the centrilobular necrosis noted on histology.^8^ When toxicity is protracted, this centrilobular pattern of necrosis may progress to more extensive confluent (bridging) necrosis as NAPQI accumulates in zone 2 and zone 1 hepatocytes.^13^ Additionally, the pro-inflammatory cytokines and chemokines released during this necrosis are thought to be responsible for the mild inflammation observed on histology.^12^

### What are the 4 clinical stages of acetaminophen toxicity?

Though not absolute for severe ingestions, acetaminophen toxicity may be divided into stages according to chronology following ingestion and certain clinical signs and symptoms:

Stage I encompasses the period beginning 30 min to 24 h after ingestion and is characterized by nausea and vomiting though many patients may be asymptomatic.^8^
Stage II encompasses days 1–3 following overdose and is characterized by hypotension and RUQ pain; it is during this stage that alanine aminotransferase (ALT) and aspartate aminotransferase (AST) elevation may first be detected.^8^
Stage III includes days 3–4 following overdose and is characterized by ALF-specific multisystem sequelae including coagulopathy, encephalopathy, metabolic acidosis, and renal failure; most deaths due to acetaminophen toxicity will occur during this stage.^8^
Stage IV describes the period after 4 days of ingestion to 3 weeks during which patients who were not deceased after stage III will experience resolution of symptoms.^8^

### Discuss the pathophysiology of the extrahepatic complications of acetaminophen-induced acute liver failure

The extrahepatic symptoms of ALF are derived from the loss of hepatic homeostatic activity and release of pro-inflammatory damage-associated molecular patterns (DAMPs) from necrotic hepatocytes. It is widely believed that the release of cytokines such as tumor necrosis factor-alpha, interleukin-1 beta, and interleukin-6 triggers a compensatory systemic inflammatory response syndrome (SIRS).^6^ Low systemic vascular tone results in vasodilation and hypotension, resulting in renal hypoperfusion (acute tubular necrosis) and cerebral hyperperfusion (cerebral edema). The latter is further enhanced by failure of hepatocytic regulation of the urea cycle, resulting in hyperammonemia, passage of ammonia through the blood-brain barrier, and increased production of glutamine through upregulation of glutamine synthetase (of which ammonia is a cofactor) in astrocytes. This increase in glutamine is significant in that glutamine's highly osmotic properties result in astrocyte swelling and depletion of ATP and GTP.^3^ The ensuing cerebral edema and hepatic encephalopathy may result in fatal uncal herniation in a subset of patients.^13^

Bacterial and fungal infections are present in 44–80% and 32% of patients with ALF, respectively.^14^ This appears to be a sequela of the compensatory anti-inflammatory response promoted by cytokines such as interleukin-4, interleukin-10, and transforming growth factor-beta.^6^ Hypoglycemia appears in many ALF patients due to absent glycogenesis and depletion of stored glycogen as well as disrupted gluconeogenesis. Metabolic acidosis may additionally develop due to insufficient blood lactic acid clearance.^6^

### What risk factors contribute to acetaminophen hepatotoxicity?

Factors predisposing individuals to acetaminophen hepatotoxicity, sometimes even at therapeutic doses of <4 g/day, include depletion of glutathione stores and CYP450 inducers. Glutathione deficiency is most often a result of malnutrition or fasting due to low cysteine intake or preexisting chronic liver disease.^10^ CYP450 (especially CYP2E1) inducers include chronic alcohol intake (this should be contrasted with acute alcohol, which has a protective effect due to CYP2E1 inhibition), anticonvulsants such as phenytoin, carbamazepine, and phenobarbital; St. John's wort; and anti-mycobacterial antibiotics such as isoniazid and rifampin. Concomitant non-alcoholic steatohepatitis (NASH) additionally increases the risk of hepatotoxicity by 4–7 times.^10^ Other factors leading to an increased risk of hepatotoxicity include unintentional overdoses (i.e. those not associated with a suicide attempt), repeated supratherapeutic injections, and genetic polymorphisms of phase II conjugative enzymes or enzymes in the CYP450 system responsible for the metabolism of acetaminophen.^10^^,^^15^

### Describe the basic approaches to treatment of acetaminophen-induced liver failure

The choice of which treatment to employ is based on the time between overdose and presentation to a medical facility. If the patient overdosed within the last 4 h, decontamination with activated charcoal and gastric lavage may be used to inhibit absorption of acetaminophen in the small intestine.^10^

Additional supportive management for ALF should consist of IV fluids and vasopressors if hypotensive, empiric proton pump inhibitors, or propranolol for variceal prophylaxis, airway protection, and maintenance of blood glucose levels.^3^ Hepatic encephalopathy may also require treatment with lactulose or select antibiotics such as rifaximin.^3^ Platelets and fresh frozen plasma are advised for ALF-associated coagulopathies.^3^

Subsequent assessment of treatment and clinical outcome often relies on the Rumack-Matthews Nomogram, a semi-logarithmic graph that measures acetaminophen levels beginning 4 h after ingestion when full absorption is expected to have taken place and ending at 24 h after ingestion. The current incarnation of the nomogram provided by the USFDA places the treatment line at 150 μg/mL.^16^ To use the nomogram, clinicians place the patient's serum acetaminophen on the nomogram at the corresponding time following ingestion – if above the 150 line at 4 h, treatment with N-acetylcysteine (NAC) should be initiated. Importantly, the nomogram should not be relied upon if the time of ingestion is unknown, acute overdose transpired over 24 hours ago, overdose was staggered rather than acute, or if repeated supratherapeutic ingestions occurred.^16^

### Explain the role of N-acetylcysteine in acetaminophen overdose treatment

NAC is a synthetic derivative of l-cysteine, one of three precursor amino acids of glutathione along with glycine and glutamate, and the precursor found in the lowest intracellular concentrations.^17^ It is for this reason, as well as its relative resistance to oxidation to a disulfide form and ability to cross phospholipid bilayers and thus more easily enter cells,^17^ that it is supplied to replenish glutathione used up in the reduction of NAPQI and neutralize accumulating ROS. In addition, NAC possesses manifold hepatoprotective mechanisms including the direct scavenging of the RNS peroxynitrite, generation of ATP-producing intermediates within the Krebs cycle,^17^ and promotion of recovery by the enhancement of hepatic perfusion and oxygen extraction.^16^

NAC may be taken orally, intravenously (IV) or inhaled, but IV is often preferred due to the drug's low oral bioavailability, especially in the acute setting.^17^ NAC is ideally given no later than 8–10 h after overdose, in which it has been demonstrated that only 6–7% will develop subsequent hepatotoxicity or ALF after administration, though it still provides hepatoprotective benefits after this period and should be administered to patients 24 h after ingestion and as a bridging treatment before liver transplants.^17^ Adverse effects include nausea, vomiting, or diarrhea as well as anaphylactic skin reactions, the latter particularly with an IV loading dose.^17^

### Discuss when liver transplants are recommended for acetaminophen hepatotoxicity

Though NAC significantly mitigates the prevalence of acute liver injury, liver transplantation is currently the only means by which to most assuredly alter patient outcomes once ALF develops. The King's College Criteria (KCC) selects patients most likely to benefit from referral to a liver transplant center. Criteria include arterial pH <7.30 after fluid resuscitation or a combination of INR >6.5 or PT >100 s, serum creatinine >3.4 mg/mL, and grade III–IV encephalopathy, findings that are collectively suggestive of multiorgan failure.^16^^,^^18^

## Diagnostic findings, Part 3

**Fig. 1:**
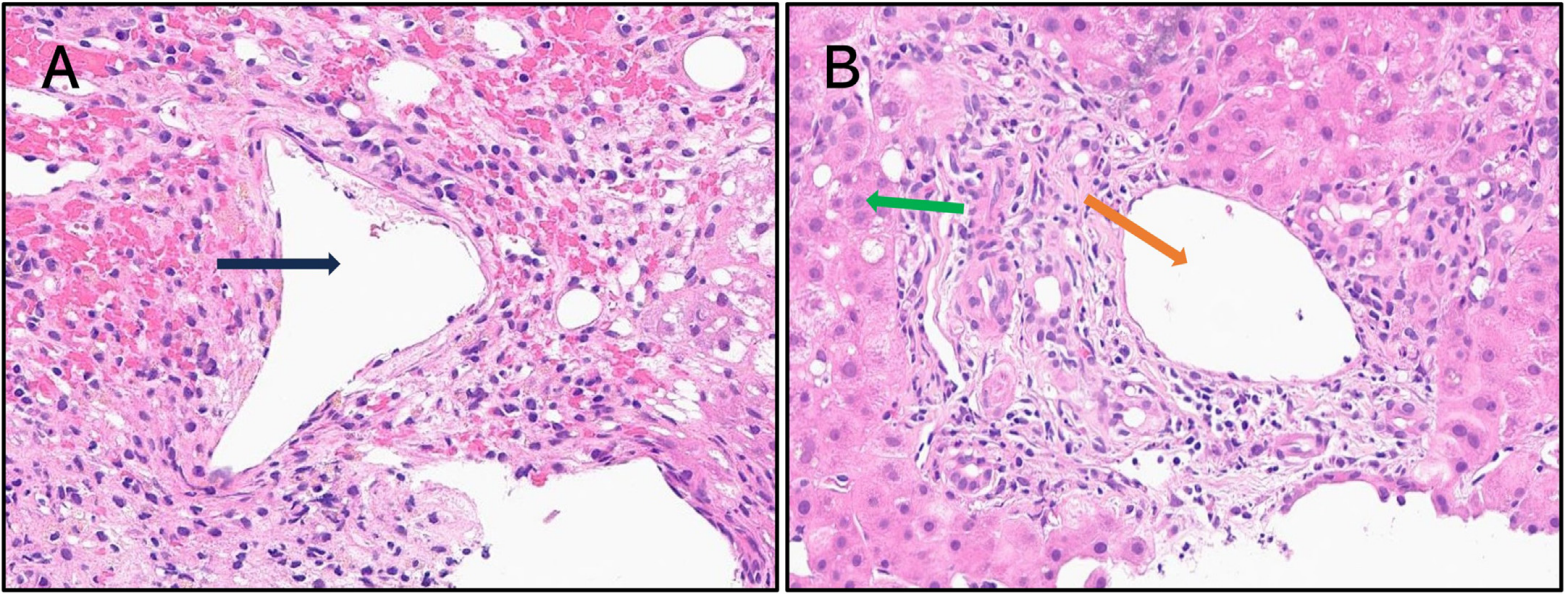
H&E-stained sections obtained from transjugular liver biopsy. **A**. Centrilobular (zone 3) necrosis is characterized by hepatocyte dropout surrounding a central vein (black arrow) with intervening congestion and mild lymphoplasmacytic infiltrates. Note the absence of viable hepatocytes (20×). **B**. Periportal (zone 1) liver parenchyma is preserved with portal vein (orange arrow) and intact hepatocytes (green arrow) shown (20×).

The patient's serum acetaminophen at 22 h post-ingestion was well above the treatment line and thus IV NAC was administered. Because the patient satisfied all of the KCC (arterial pH 7.25, INR 6.7, serum creatinine 3.8, and grade III and IV encephalopathy), it was expected that this patient would suffer a poor prognosis and thus referral for transplant was promptly initiated with NAC maintained as a bridging treatment. A transjugular liver biopsy from the right lobe was submitted to assess liver adequacy with H&E-stained microimages depicted in Fig. 1.

## Questions/discussion points, Part 3

### Describe the histologic findings of this patient's liver

The patient's liver displays a centrilobular (also called zone 3 or perivenular) pattern of hepatocyte necrosis characterized by hepatocyte dropout and ballooning degeneration surrounding a central vein (see Fig. 1A). Passive congestion and mild lymphoplasmacytic inflammation are noted due to the release of pro-inflammatory cytokines during necrosis. Corroborating the pure centrilobular necrosis present throughout the patient's liver, hepatocytes surrounding the portal tract (zone 1) are preserved with no evidence of necrosis (see Fig. 1B). Though not present in this liver specimen, confluent necrosis in which larger swathes of hepatocytes undergo dropout and may bridge adjacent centrilobular zones may be present.^13^

### What are other potential causes of centrilobular necrosis?

Centrilobular necrosis is most commonly a result of DILI. Of the commonly reported etiologies, drugs that exert their hepatotoxic effects through dose-dependent mechanisms are most frequent and include drugs such as cocaine and tetracycline.^19^ Other dose-dependent causes of centrilobular necrosis include ingestion of toxins such as carbon tetrachloride, chloroform, aflatoxin B produced by *Aspergillus flavus*, and pyrrolizidine alkaloids found within select plant species and the herbivorous animals who consume them.^19^ Idiosyncratic reactions rarely induce centrilobular necrosis but some examples may include the anesthetics enflurane and halothane (which can also present as periportal necrosis), isoniazid, and valproate.^13^

Ischemia secondary to shock or heart failure classically presents as zone 3 necrosis with a mild inflammatory infiltrate not unlike that seen in acetaminophen toxicity. Hepatic vein thrombosis (Budd-Chiari Syndrome), in addition to centrilobular necrosis, typically presents with prominent sinusoidal dilation in the acute setting. Finally, reperfusion injury following management of ischemia may present as zone 3 necrosis.^13^

### Discuss the prognosis of patients who develop acetaminophen toxicity

The mortality of acetaminophen hepatoxicity is high if treatment is not initiated, but prompt treatment initiation and increased recognition of the clinicopathologic picture of this toxicity has resulted in a mortality of <2%.^8^^,^^10^ All of the aforementioned KCC are associated with increased mortality, but metabolic acidosis appears to be the most prominent criterion as untreated mortality may be as high as 95%.^18^ Other factors associated with mortality include chronic alcoholism as well as the concomitant use of medications such as fibrates and non-steroidal anti-inflammatory drugs (NSAIDs)^10^ and staggered overdoses, which though associated with lower serum acetaminophen and ALT on admission as well as lower total acetaminophen intake, often confers poor odds of survival.^20^ Favorable outcomes are associated with age less than 6 years old, as the phase II conjugative enzymes of children harbor a higher saturation point,^8^ and elevated serum alpha-fetoprotein (AFP), a marker of hepatic recovery in patients with ALF.^11^

## Conclusion

Following liver transplantation, the patient recovered to their pre-ingestion baseline status.

## Teaching points

• Acetaminophen hepatotoxicity most commonly presents as ALF characterized by signs and symptoms of acute liver injury (RUQ pain, jaundice, loss of appetite, etc.) as well as multiorgan failure characterized by renal failure, encephalopathy, and coagulopathy (INR > 1.5).
• Acetaminophen is the most common cause of ALF in developed countries and usually presents in a rapid (hyperacute) fashion compared to other etiologies.
• The pathophysiology of acetaminophen hepatotoxicity is due to the accumulation of excess toxic NAPQI in hepatocytes, mitochondrial injury, and eventual hepatocyte necrosis.
• Risk factors of acetaminophen hepatotoxicity include the depletion of glutathione stores (malnutrition or fasting), CYP450 inducers, unintentional overdoses, and genetic polymorphisms of phase II conjugative enzymes.
• Early treatment (<4 h post-ingestion) consists of decontamination with activated charcoal or gastric lavage.
• N-acetylcysteine replenishes glutathione stores and is the treatment of choice for acetaminophen hepatotoxicity; administration between 4 and 24 h often relies on assessment of serum acetaminophen levels via the Rumack-Matthew Nomogram.
• Liver transplantation is recommended for patients who meet the KCC: Arterial pH <7.30 and/or a combination of grade III–IV encephalopathy, serum creatinine >3.4 mg/mL, and INR >6.5.
• Histologically, acetaminophen hepatotoxicity presents as centrilobular (zone 3) necrosis, owing to the high concentration of CYP450 enzymes within this region, or panacinar necrosis with minimal inflammation.
• The prognosis of acetaminophen-induced ALF is favorable post-treatment.

## Funding

The article processing fee for this article was funded by an Open Access Award given by the Society of '67, which supports the mission of the Association for Academic Pathology to produce the next generation of outstanding investigators and educational scholars in the field of pathology. This award helps to promote the publication of high-quality original scholarship in *Academic Pathology* by authors at an early stage of academic development.

## Declaration of competing interest

The authors declare that they have no known competing financial interests or personal relationships that could have appeared to influence the work reported in this paper.
